# Subtyping of *Blastocystis* sp. isolated from symptomatic and asymptomatic individuals in Makkah, Saudi Arabia

**DOI:** 10.1186/s13071-017-2114-8

**Published:** 2017-04-07

**Authors:** Raafat T. Mohamed, Mohammed A. El-Bali, Anhar A. Mohamed, Mona A. Abdel-Fatah, Mohamed A. EL-Malky, Nawras M. Mowafy, Dina A. Zaghlool, Rowaida A. Bakri, Saeed A. Al-Harthi

**Affiliations:** 1 Department of Medical Parasitology, Faculty of Medicine, Umm Al-Qura University, Makkah, Saudi Arabia; 2 Department of Medical Parasitology, Faculty of Medicine, El-Minia University, El-Minia, Egypt; 3 Department of Medical Parasitology, Faculty of Medicine, Ain Shams University, Cairo, Egypt; 4 Microbiology Laboratory, King Abdellah Medical City, Makkah, Saudi Arabia; 5 Department of Medical Parasitology, Faculty of Medicine, Mansoura University, Mansoura, Egypt; 6 Laboratory and Blood Bank Department, Al-Noor Specialist Hospital, Makkah, Saudi Arabia; 7 Department of Medical Parasitology, Faculty of Medicine, Assiut University, Assiut, Egypt

**Keywords:** *Blastocystis* sp, Sequence-Tagged Sites (STS) PCR, Subtyping, Makkah city

## Abstract

**Background:**

*Blastocystis* is a group of cosmopolitan gastrointestinal parasite of humans and a wide variety of animals. These anaerobic protozoans include more than 17 specific small-subunit ribosomal RNA subtypes, of which nine are found in humans with a variable geographical distribution. Until now, no study has described the *Blastocystis* subtypes present in Saudi Arabia.

**Methods:**

In total, 1,262 faecal samples were collected from patients with gastrointestinal complaints and asymptomatic individuals visiting two major hospitals. All samples were analysed by F1/R1 diagnostic PCR, microscopy and culture methods. The subtypes of *Blastocystis* sp. isolates were determined by the sequenced-tagged site (STS)-based method.

**Results:**

One-hundred-thirty-three positive cases were detected by F1/R1 diagnostic PCR, of which 122 were also positive by the culture method and 83 by direct microscopy. The sensitivities of direct microscopy and the culture method were 62% and 92%, respectively. Subtype (ST3) was the most prevalent (80.5%), followed by ST1 (14.5%) and ST2 (5%). ST4, ST5, ST6 and ST7 were not detected in this study. ST3 infections were significantly predominant (*P* < 0.05) among symptomatic patients.

**Conclusions:**

To our knowledge, this study provides the first run-through information on *Blastocystis* sp. epidemiology in Makkah city, revealing a rather moderate prevalence of 10.5% and the presence of three subtypes, ST1, ST2, and ST3. ST3 was the most predominant, particularly among symptomatic patients.

## Background

*Blastocystis* is a group of cosmopolitan gastrointestinal anaerobic protozoan parasites. The group is known to infect humans and a wide variety of animal hosts, including mammals, birds, amphibians, and reptiles [1]. The life-cycle comprises numerous ‘forms’, including granular cysts and multivacuolar, avacuolar, vacuolar, and amoeboid structures. The mode of infection has not been completely understood until now, but involves faecal-oral ingestion of a cyst, with animal handlers showing significantly high rates of infection [2, 3]. *Blastocystis* has a worldwide distribution with a marked prevalence in many countries. According to most epidemiological studies, nearly all countries of the world have been classified into well-developed, with a moderate prevalence (10-15%), or under-developed, with a high prevalence (55–70%), attributed to the levels of hygiene and the presence or absence of contact with animals and/or contaminated water and food [4, 5].

*Blastocystis* may exist in the gut for years; however, the recognition of a high prevalence of *Blastocystis* in healthy populations using sensitive molecular diagnostic tools has led to a standard shift in clinical *Blastocystis* research. Studies of the gut microbiota in individuals with and without *Blastocystis* can provide important information to help determine the role of *Blastocystis* in human health and disease [6]. Additionally, the *Blastocystis* parasite appears to be more common in healthy individuals than in patients with inflammatory bowel disease and is associated with certain gut microbiota and health indicators. Although the parasite may elicit disease under certain conditions, the focus on *Blastocystis* may be shifting from a clinical to a public health viewpoint [7]. SSU rRNA-based phylogeny and host origins do not associate with each other for the classification of different *Blastocystis* isolates that are derived from humans to insects. The *Blastocystis* sp. subtype (ST) classification is applied for *Blastocystis* isolates from homoeothermic animals because their origin is from humans, and they are classified into nine genetically different STs. However, the situation in poikilothermic animals, such as reptiles and amphibians, suggests difficulties; therefore, determining the complete sequence of SSU rDNA is essential to establish novel ST in the phylogenetic analysis [8].

In *Blastocystis* sp. infection, the symptoms are mostly nonspecific and include weight loss, nausea, vomiting, flatulence, abdominal pain, and diarrhoea [9, 10]. However, the pathogenicity of this parasite is still argumentative and non-conclusive; *Blastocystis* sp. infections have been reported in several countries in many asymptomatic and apparently healthy individuals as well as in symptomatic cases without any obvious causal organisms [2]. Several studies have deduced that only a few subtypes of *Blastocystis* sp. can cause symptoms. It was concluded that the proteases of *Blastocystis* sp. subtype 3 (ST3) were responsible for protein degradation with host immune evasion and are an important virulence factor [11, 12]. Another study correlated *Blastocystis* sp. subtype 3 infections with idiopathic urticaria [12].

In last few years, several molecular tools have been used to classify the *Blastocystis* sp. subtypes isolated from humans and animals in different regions worldwide. These techniques include random amplified polymorphic DNA (RAPD) using four different arbitrary polymerase chain reaction (PCR) primers [13] and PCR assays followed by restriction fragment length polymorphism (RFLP) to study the sequence variation in the small-subunit ribosomal RNA genes of isolates [14]. More recently, new PCR-based methods have been developed for subtype classification using known sequenced-tagged site (STS) primer sets [15] in combination with single-strand conformational polymorphism (SSCP) analysis [1] or post-PCR pyrosequencing techniques based on the detection of subtype-specific nucleotide polymorphism in the SSU rRNA gene [16, 17], fingerprinting with AP-PCR [18], and multilocus sequence typing [19]. The latest epidemiological studies have shown the great diversity of *Blastocystis* sp. subtypes; ten subtypes (ST1 to ST10) have been found according to SSU rRNA gene sequences [20]. Seven other subtypes (ST11–ST17) have been identified in isolates from livestock and zoo animals [21]. Among all identified subtypes, only ST1 to ST9 are considered to be human *Blastocystis* sp. subtypes [10, 22]. Some authors recommend a standardization of *Blastocystis* terminology to simplify statements and show the relationships of the research results. This primarily depends on the already published data on small-subunit ribosomal RNA gene analyses and suggests that all mammalian and avian isolates should be nominated as *Blastocystis* sp. [23]. Using *Blastocystis* genetic markers, the barcode region is the best region to use in publicly available sequence databases at GenBank, while the STS primers represent a suitable approach in situations where sequencing is not an option, and the disadvantages of using STS primers are that some subtypes will go undetected, includes ST8 and ST9 and also the majority of ST4 strains [24].

To our knowledge, no previous study has explored the prevalence and subtype distribution of *Blastocystis* sp. in Saudi Arabia. The aim of the present study was to explore the *Blastocystis* sp. relative prevalence and subtypes present in one of the major cities in Saudi Arabia. This survey was performed on faecal samples collected from symptomatic and asymptomatic individuals who visited, over a 10-month period, two major hospitals of Makkah city, Saudi Arabia.

## Methods

### Sample collection

This study was carried out from March 2014 to January 2015 at two major health care centres of Makkah city, King Abdellah medical city and Al-Noor Specialist Hospital. Of all faecal specimens received during this period by Parasitology Laboratory units, in both hospitals, 1,262 specimens were included in this investigation. Approximately 50% of the collected samples were from patients with gastro-intestinal complaints, including severe abdominal pain and diarrhoea, and the other 50% were from apparently healthy individuals who underwent a regular check-up or mandatory health check.

### Microscopic examination and in vitro culture

The referred faecal specimens are routinely examined by microscopy for parasitic infections using the direct smear method with saline and iodine. Samples from suspected patients, if negative by direct microscopy, are further examined after trichrome permanent staining and according to the formalin ethyl acetate concentration. For this study, each collected sample was split into two parts; one part was kept at -20 °C, and the other part was inoculated in culture media and transferred to the medical parasitology laboratory of the Faculty of Medicine at Umm Al Qura University for sub-culturing and molecular analysis. The culture medium consisted of Dulbecco's modified Eagle medium (DMEM) (Gibco, Thermo Fisher Scientific, MA, USA) containing 12 mg/ml ampicillin and 4 mg/ml streptomycin supplemented with 20% inactivated horse serum (Gibco) sterilized by filtration [25]. The samples were cultured in 11 × 100 mm sterile screw-capped tubes containing 3 ml of media and were incubated at 37 °C in an anaerobic gas pack (BD gas pack-Becton, Dickinson, USA). A drop of culture was examined after 72 h by direct microscopy.

### DNA extraction

Genomic DNA was extracted directly from stool samples using the ZR faecal DNA miniprep kit (Zymo Research, Tustin, CA, USA) and from cell pellets of positive cultures using the QIAmp DNA extraction kit (QIAmp, QIAGEN, Inc., Hilden, Germany), according to manufacturers’ protocols. The concentration and purity of isolated DNA were measured by a spectrophotometer (SpectraDrop; SpectroMax, Life Technology, Carlsbad City, CA, USA).

### F1/R1 diagnostic PCR

Diagnostic PCR was applied to genomic DNA extracted from stool specimens using the F1 (5'-GGA GGT AGT GAC AAT AAA TC-3') and R1 (5'-CGT TCA TGA TGA ACA ATT AC-3') primers [26]. Briefly, 2 μl of genomic DNA was used in PCR reactions of a 25 μl final volume using the AmpliTaq Gold 360 master mix (Applied Biosystems, CA, USA). The PCR conditions consisted of one cycle of initial denaturation at 94 °C for 4 min, followed by 35 cycles of denaturation at 94 °C for 30 s, annealing at 54 °C for 30 s, extension at 72 °C for 30 s, and a final elongation cycle for 5 min at 72 °C.

### *Blastocystis* sp. subtyping using Sequence-Tagged Site (STS) primers

PCR reactions were carried out using sequence-tagged site primer sets derived from the RAPD product sequence to identify the subtypes as follows: ST1 variant (SB82: 5'-TCT TGC TTC ATC GGA GTC / CCT TCT CGC AGT TCT TTA TC-3'), ST1 (SB83: 5'-GAA GGA CTC TCT GAC GAT GA / GTC CAA ATG AAA GGC AGC-3'), ST7 (SB155: 5'-ATC AGC CTA CAA TCT CCT C / ATC GCC ACT TCT CCA AT-3'), ST3 by multiplex PCR including (SB227: 5'-AGG ATT TGG TGT TTG GAG A / TTA GAA GTG AAG GAG ATG GAA G-3', SB228: 5'-GAC TCC AGA AAC TCG CAG AC / TCT TGT TTC CCC AGT TAT CC-3' and SB229: 5'-CAC TGT GTC GTC ATT GTT TTG / AGG GCT GCA TAA TAG AGT GG-3'), ST6 (SB332: 5'-GCA TCC AGA CTA CTA TCA ACA TT / CCA TTT TCA GAC AAC CAC TTA-3'), ST5 (SB336: 5'-GTG GGT AGA GGA AGG AAA ACA / AGA ACA AGT CGA TGA AGT GAG AT-3'), ST4 (SB337: 5'-GTC TTT CCC TGT CTA TTC TTG CA / AAT TCG GTC TGC TTC TTC TG-3'), and ST2 (SB340: 5'-TGT TCT TGT GTC TTC TCA GCT C / TTC TTT CAC ACT CCC GTC AT-3') [15]. Two microlitres of DNA extract were used in PCR reactions of a 25 μl final volume using the AmpliTaq Gold 360 master mix (Applied Biosystems, Carlsbad City, CA, USA). The PCR conditions consisted of one cycle of initial denaturation at 94 °C for 5 min, followed by 40 cycles that included denaturation at 94 °C for 30 s, annealing at 57 °C for 30 s, extension at 72 °C for 1 min, and a final elongation cycle for 5 min at 72 °C. All PCR amplifications were carried out in duplicate for each sample and each primer set. The PCR products were separated in 1.5% agarose gels and were photographed.

### Statistical analysis

The data were analysed using the Chi-square test to compare the frequency of the *Blastocystis* sp. subtypes from symptomatic and asymptomatic individuals. A *P*-value < 0.05 was statistically significant. Statistical analysis was performed using SPSS version 21.

## Results

### Relative prevalence of *Blastocystis* sp. based on microscopy and in vitro culture

Of 1,262 examined faecal samples, 133 specimens were found to be positive for *Blastocystis* by the F1/R1 diagnostic PCR technique used in this study. Among positive cases, 85 (64%) were patients with gastrointestinal symptoms and 48 (36%) were asymptomatic individuals. The general symptoms consisted of abdominal pain in approximately 76%, diarrhoea in approximately 27% and other gastrointestinal complaints in approximately 16% of the symptomatic patients. All 133 positive cases were detected by F1/R1 diagnostic PCR, of which 122 were also positive by the culture method and 83 by direct microscopy. The sensitivities of direct microscopy and the culture methods compared with that of F1/R1 PCR and are shown in Table 1.

**Table 1:** Sensitivity of F1/R1 PCR, direct microscopy, and the culture method for the diagnosis of *Blastocystis* sp. infections in the stool samples of patients with GI symptoms and asymptomatic individuals

Diagnostic technique	Symptomatic (*n* = 85)	Asymptomatic (*n* = 48)	Total (*n* = 133)	Sensitivity
Direct microscopy	65	18	83	62%
In vitro culture	79	43	122	92%
F1/R1 PCR	85	48	133	100%

### STS subtyping analysis of *Blastocystis* sp. isolates

**Fig. 1:**
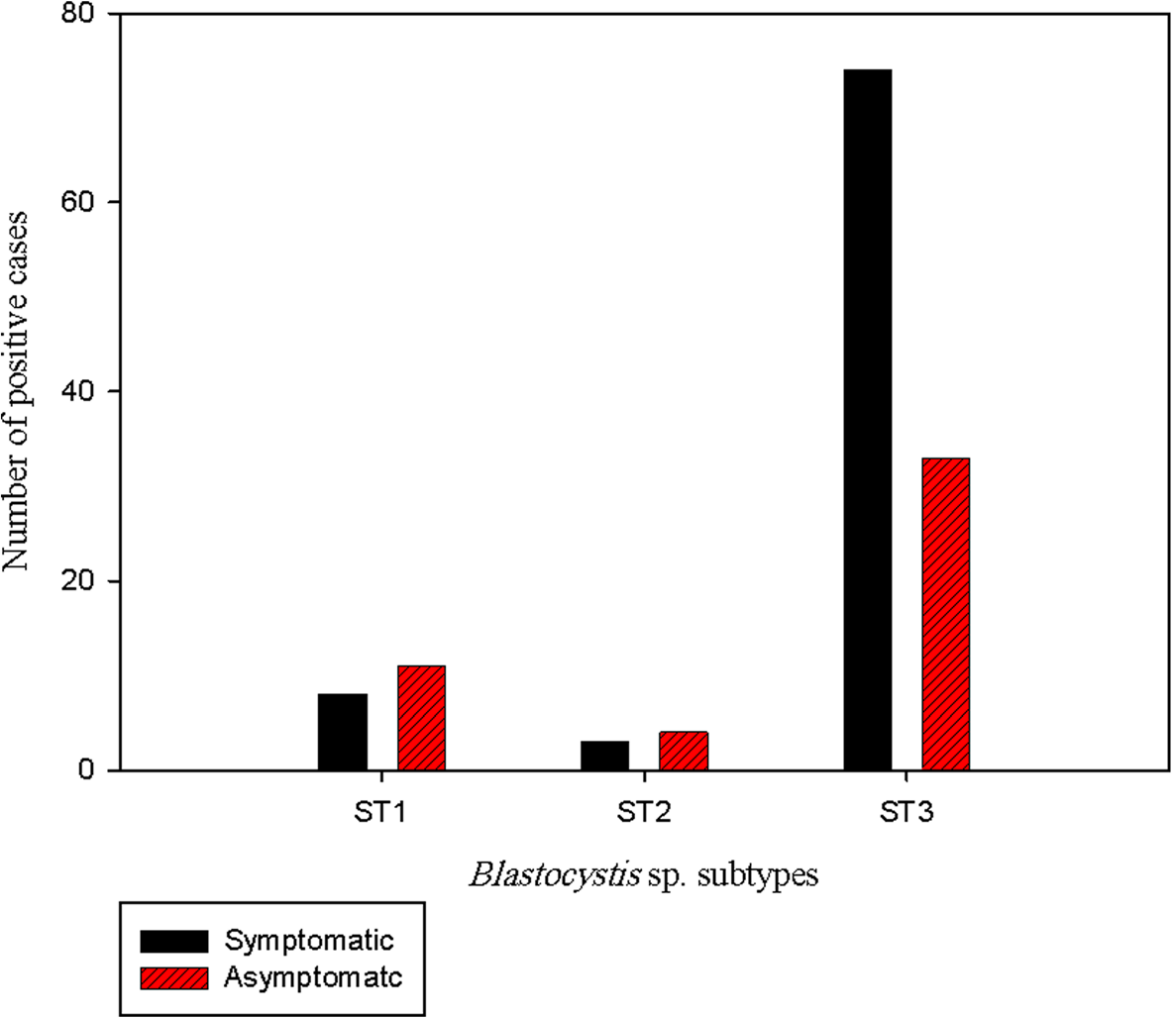
Subtype distribution of *Blastocystis* sp. among gastrointestinal patients and asymptomatic individuals. Subtype 3 was 69% in symptomatic patients and 31% in asymptomatic individuals (*χ* ^2^ = 6.54, *df* = 2, *P* < 0.05)

Three *Blastocystis* sp. subtypes (ST1, ST2 and ST3) were detected by STS primer-based PCR analysis of positive samples. ST3 was the most prevalent (80.5%), followed by ST1 (14.5%) and ST2 (5%). ST4, ST5, ST6 and ST7 were not detected in this study. ST3 infections were significantly predominant (*χ* ^2^ = 6.54, *df* = 2, *P* = 0.038) among symptomatic patients (Fig. 1). The *Blastocystis* sp. subtype distribution did not show a predisposition in relation to the age or gender of infected individuals.

## Discussion

In this study, the relative prevalence of *Blastocystis* sp. infection among individuals with or without gastrointestinal symptoms who visited two major hospitals in Makkah city was determined to be 10.5%. Since the prevalence data are usually derived from general surveys of intestinal parasites using the formol acetate concentration technique with microscopic examination, such numbers should be interpreted with extreme caution because the sensitivities of this method are lower than those of nucleic acid-based methods, including conventional and real-time PCR [7]. Epidemiological studies in several countries with different sanitation standards revealed wide ranges of *Blastocystis* sp. prevalence: 0.54% in Turkey [27], 2.5% in Japan [15], 3.3% in Singapore [28], 19% in Lebanon [29], 22.1% in Libya [30], 33.3% in Egypt [31], 40.7% in the Philippines [32] and 46.9% in Venezuela [33]. Thus, the prevalence found herein is relatively moderate compared with the worldwide general infection situation.

Numerous conflicting reports on the pathogenic capability of *Blastocystis* sp. infection have been published, and it is still under debate whether *Blastocystis* sp. is a pathogenic or a commensal life form [2]. In our study, *Blastocystis* infections were detected both in patients with patent gastrointestinal symptoms (85 cases) and in apparently healthy individuals (48) who underwent mandatory health check-ups. Additionally, 78.5% of symptomatic patients and 21.5% of asymptomatic individuals with PCR-positive infections were positive by microscopy; accordingly, it has been reported that the *Blastocystis* cell density is usually significantly higher in symptomatic patients [34]. Comparative studies of human immunity against *Blastocystis* infection have revealed a significant difference in antibody responses between symptomatic and asymptomatic individuals [35, 36]. However, antigenic heterogeneity has been detected between *Blastocystis* sp. isolated from symptomatic patients and asymptomatic individuals in axenic culture; however, these studies did not explore any possible correlations between this heterogeneity and genetic subtypes [37].

Nine subtypes of *Blastocystis* sp. (ST1 to ST9) were identified in humans [10]. To our knowledge, this is the first report exploring the subtypes of *Blastocystis* in Makkah city, Saudi Arabia. Using sequence-tagged site (STS) primer-based PCR, we found three *Blastocystis* sp. subtypes (ST1, ST2 and ST3) among the 133 positive cases. The high predominance of ST3 (80.5%) in our study population agrees with different reports in the literature; ST3 was found to be the most dominant subtype, varying from 41% to 92%, in a comparative study between Japan, Bangladesh, Pakistan and Germany [15] and at 78%, 75.9%, 54.5%, 53.5% and 33.3% in Singapore, Turkey, Egypt, France and Lebanon, respectively [28, 29, 38–40]. However, the ST1 (50%) subtype was found to be more predominant than ST3 (39.5%) in the Libyan population [41], as confirmed by a more recent study reporting the predominance of ST1 (51.1%) over ST3 (17.8%) in Libya [30]. Although ST2 is commonly reported to be the second most prevalent subtype [42–45], in our study, only 5% of cases carried the ST2 subtype, while 14.5% carried ST1.

Subtypes ST4, ST5, ST6 and ST7, as reported in previous studies [39, 46, 47], were not detected in our investigation. ST4 was remarkably common in Danish patients with acute diarrhoea [48], symptomatic patients in Spain [43], and rural communities in Nepal [49]. ST6 and ST7 were reported in the Middle East both in irritable bowel syndrome patients and healthy persons [39, 47]. However, ST5 was mostly reported in animals and humans living near to farms [16, 50].

In many previous studies, no association was reported between the specific subtype of *Blastocystis* sp. and explicit gastrointestinal symptoms [40, 46, 51]. However, other studies reported a specific association between subtypes of the parasite and apparent symptoms: it was demonstrated that *Blastocystis* cysteine proteinase can degrade human IgA [52]. Additionally, patients with chronic gastrointestinal illness with reported antibiotic failure for over four years were diagnosed with ST3 and ST1 infections [53]. More recently, irritable bowel syndrome in several Mexican patients was associated with *Blastocystis* sp. ST1 and ST3 infections [54]. Consistently, ST3 infections were significantly predominant among symptomatic patients (69%) in our study. *Blastocystis* sp. ST2 infection was linked to gastrointestinal illness and chronic urticaria [55].

In our findings, this subtype did not show an apparent predominance in distribution between symptomatic and asymptomatic infected individuals. However, it appears that the *Blastocystis* sp. infection clinical outcome is multifactorial, complicating the evaluation of its pathogenicity, even during case-controlled studies [5].

## Conclusions

The outcome of this study provides the first run-through information on *Blastocystis* sp. epidemiology in Makkah city, revealing a relatively moderate prevalence (10.5%), as well as the presence of three subtypes: ST3, the most predominant particularly among symptomatic patients; ST1; and ST2. Further screenings are needed to clarify the epidemiology of this gastrointestinal parasite in local and emigrant populations of the entire country. Likewise, the present study underlines the advantage of STS-PCR as a significant technique for *Blastocystis* sp. subtyping in epidemiological studies.

## Abbreviations

AP-PCR: Arbitrary primer polymerase chain reaction
DMEM: Dulbecco's modified Eagle medium
RAPD: Random amplified polymorphic DNA
RFLP: Restriction fragment length polymorphism
SSCP: Single strand conformational polymorphisms
SSU rRNA: Small-subunit of the ribosomal RNA
STS: Sequence-tagged site
STs: Subtypes
